# Case report: Shock after percutaneous vertebroplasty of the 5th thoracic vertebra

**DOI:** 10.3389/fsurg.2023.1120346

**Published:** 2023-05-30

**Authors:** Shenshen Hao, Xin Yu, Laihao Li, Shuai Liu, Hongke Li, Shengli Dong, Xinhao Cao

**Affiliations:** ^1^ Department of Spine and Bone Oncology, General Hospital of Pingmei Shenma Medical Group, Pingdingshan City, China; ^2^ Emergency Department, Xi'an Honghui Hospital, Xi'an City, China

**Keywords:** percutaneous vertebroplasty, osteoporotic vertebral compression fracture, shock, treatment, case report

## Abstract

**Background:**

Percutaneous vertebroplasty (PVP) is a common treatment for osteoporotic vertebral compression fracture (OVCF). Perioperative bleeding is usually rare, so there are few reports of shock. However, we developed shock after treating a case of OVCF of the 5th thoracic vertebra with PVP.

**Case presentation:**

An 80 years old female patient received PVP due to OVCF of the 5th thoracic vertebra. The operation was successfully completed and the patient returned to the ward safely after the operation. At 90 min after operation, she developed shock, which was induced by subcutaneous hemorrhage up to 1500 ml at the puncture site. Before using vascular embolization, transfusion and blood transfusion were used to maintain blood pressure, and local ice bag compression was used to reduce swelling and stop bleeding, which achieved successful hemostasis. She recovered and discharged after 15 days, with the hematoma having absorbed. There was no recurrence during the 17-month follow-up.

**Conclusion:**

Although PVP is considered to be a safe and effective method to treat OVCF, the possible hemorrhagic shock still needs to arouse the vigilance of surgeons.

## Introduction

Osteoporotic vertebral compression fracture (OVCF) is the most common fracture injury caused by osteoporosis in the elderly (1). Percutaneous vertebroplasty (PVP) is one of the most commonly recommended methods for treatment of OVCFs (2, 3). It can quickly and efficiently relieve pain, fix fractured vertebra and strength spinal stability (4–7). Meanwhile, it is also a minimally invasive operation with simple operation, small trauma and fast recovery.

Generally, the common complications of PVP include bone cement leakage, cement embolism, intraoperative puncture injury and new fractures (8–11). Bone cement leakage and embolism may cause serious adverse complications, such as pulmonary cement embolism (12–15), spinal compression (16, 17), neurological deficit (18, 19), atrial mass cement embolism (20), and iatrogenic venous compression (21), which have attracted a lot of attention. However, there are few reports about the hemorrhage caused by vascular injury, although the bleeding may lead to shock or other serious and terrible complications.

We developed shock after treatment of OVCF of the 5th thoracic spine with PVP. In view of its rarity, we reviewed the literature and found that only two cases of massive hemorrhage after PVP were reported (22, 23). Moreover, both cases occurred in the lumbar spine and were treated by arterial embolization. In our case, she was well treated by non-surgical intervention. However, there is no report of massive hemorrhage of thoracic vertebrae after PVP. Given the danger of shock, we report this case. We have two purposes. One is to discuss the possible causes and treatment of postoperative bleeding. The other is to remind surgeons to be alert to the possibility of shock caused by postoperative hemorrhage.

## Case presentation

**Figure 1:**
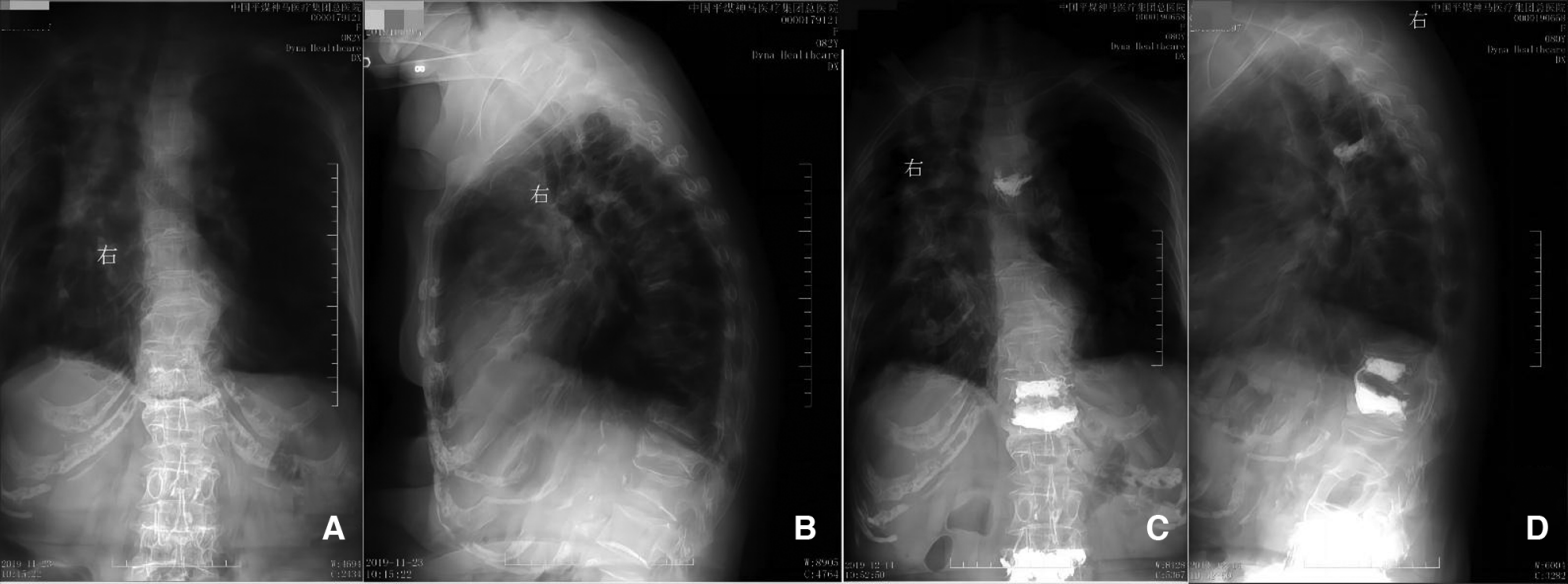
Preoperative (panels **A** and **B**) and postoperative (panels **C** and **D**) radiographs. Panels **A** and **B** correspond to the preoperative anterior and lateral positions, respectively, indicating compression changes in the 5th thoracic vertebra. Panels **C** and **D** correspond to the 12th day postoperatively demonstrating anterior and lateral positions respectively, showing the cemented 5th thoracic vertebra.

An 80-year-old female, height 155 cm, weight 45 kg, was admitted to our department with chief complaint of “lower back pain and limited mobility for more than 10 days”. The patient had lower restricted movement and back pain for unobvious reasons. The pain aggravated while she was turning over or waking up, but it was not relieved after conservative treatment. The patient had a history of pulmonary tuberculosis healed after regular anti-tuberculosis treatment, and had not taken medicine for a long time. Besides, the patient had no history of blood system disease or long-term anticoagulation therapy. She had a history of osteoporosis and multiple old spinal fractures. In recent years, the patient gradually appeared hunchback. Physical examination revealed slight kyphosis of the thoracolumbar spine and restricted range of motion. There was tenderness and percussion pain in the back. Both Lasegue's signs and Babinski's signs were negative. No edema was in the lower limbs and the peripheral blood supply was normal. Magnetic resonance imaging (MRI) revealed OVCFs in the 5th and 12th thoracic vertebrae and the 1st and 4th lumbar vertebrae, with multiple old vertebral fractures. Besides, radiographies illustrated compression of spinal vertebrae (Figures 1A,B). Laboratory examinations showed that white blood cell (WBC) was 8.02 × 10^9^/L, red blood cell (RBC) 3.78 × 10^12^/L, hemoglobin (HB) 115 g/L, platelet (PLT) 205 × 10^9^/L, erythrocyte sedimentation rate (ESR) 28 mm/h, C-reactive protein (CRP) 55.00 mg/L. Thromboelastography results showed that coagulation factor level, fibrinogen function, platelet function and fibrinolysis system were normal, with R value at 5.8 min, K value at 1.6 min, Angle value at 67.8 degree, MA value at 61.5 mm, LY30 value at 0%, EPL value at 0% and CI value at 0.5. These suggested that the coagulation function were normal. Bence-Jones protein qualitative test result was negative. T-SPOT (tuberculosis) test result was negative. Our initial diagnosis included 1. OVCF (T5/T12/L1/L4), 2. Old spinal fracture, and 3. A history of pulmonary tuberculosis.

**Figure 2:**
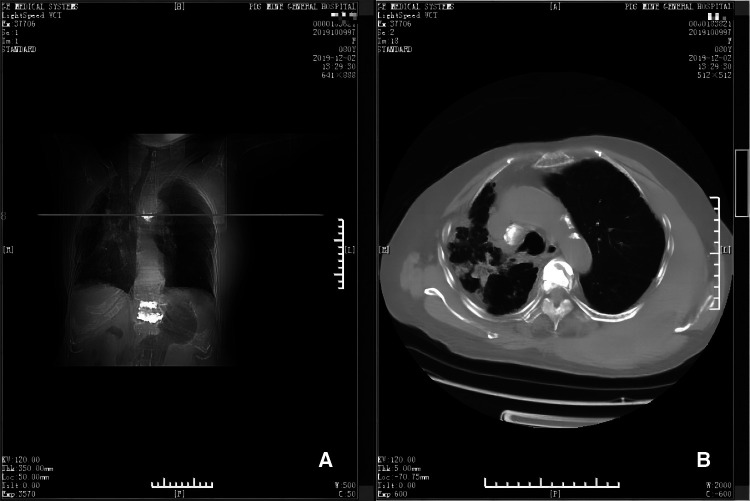
Emergency CT scans (panels **A** and **B**). Panel **A** depicts the position (horizontal line) of the CT scan shown in **B**, at the level of the 5th thoracic vertebra. Panel **B** shows that there is a large hematoma near the 5th thoracic vertebra and adjacent ribs.

The patient received anti-inflammatory treatment due to the high inflammatory index before operation. There were 4 OVCF requiring surgical treatment, and they were divided into two operations. The first surgical plan including three OVCFs (T12/L1/L4) was successfully completed, and the postoperative vital signs were stable. A symptomatic treatment was conducted, including infection prevention, analgesia, local ice bag compression and so on. There was no swelling or hematoma at the surgical sites. PVP for the OVCF (T5) was performed on the 2nd day after the initial surgery before postoperative radiographies were taken (Figures 1C,D). During the operation, a mild local swelling was found at the operation site, with minor bleeding. Therefore, an local ice bag compression was applied to stop the bleeding for 30 min, and the bleeding was successfully stopped, with blood pressure at 98/60 mmHg. However, about 90 min after surgery, the patient developed shock. She fainted for a few seconds, and the blood pressure gradually dropped to 62/36 mmHg, accompanied by obvious swelling of his back. She was immediately given a rapid infusion of liquid to raise her blood pressure, and at the same time, 400 ml of blood including 2 units of red blood cells was transfused. Meanwhile, local ice bag compression was applied to stop the bleeding and detumescence. The emergency blood tests indicated that WBC was 11.26 × 10^9^/L, RBC 2.95 × 10^12^/L, HB 84 g/L, PLT 262 × 10^9^/L, CRP 11.00 mg/L, prothrombin time (PT) 12.00 s, activated partial thromboplastin time (APTT) 31.20 s, thrombin time (TT) 20.07 s, fibrin/fibrinogen degradation products (FDP) 12.67 µg/ml, potassium (K^+^) 3.20 mmol/L, sodium (Na^+^) 138 mmol/L, and chloride (Cl^−^) 106 mmol/L. Additionally, an emergency computed tomography (CT) scan revealed that an irregular soft tissue mass with uneven density on the left chest wall, with a subcutaneous hemorrhage amounted to approximately 1500 ml, and without evidence of bone destruction or hematoma in ribs adjacent to the 5th thoracic vertebra (Figure 2).

**Figure 3:**
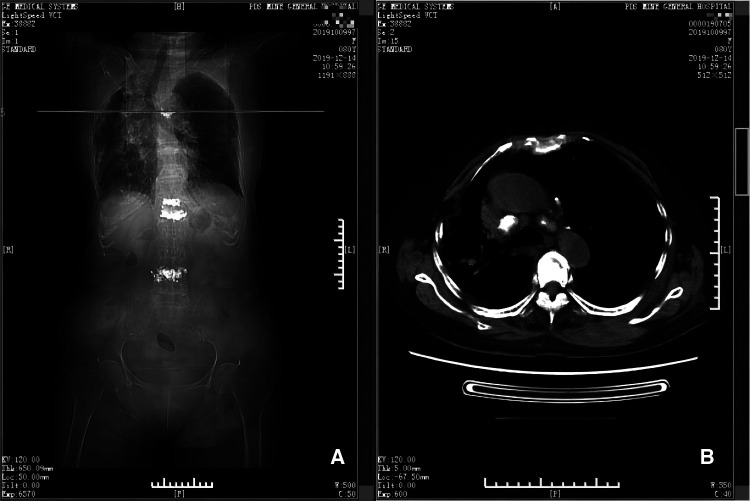
CT scan taken on 12th day postoperatively (panels **A** and **B**). Panel **A** depicts the position (horizontal line) of the CT scan shown in **B**, at the level of the 5th thoracic vertebra. Panel **B** shows there is little remaining hematoma near the 5th thoracic vertebra and adjacent ribs.

The patient was in hemorrhagic shock. After the emergency consultation, a symptomatic treatment was given, which including 400 ml of blood transfusion (containing 2 units of red blood cells), 500 ml of glucose and sodium chloride rehydration (containing 25 g glucose and 4.5 g sodium chloride) and 30 ml of oral potassium chloride supplement (containing 3 g potassium chloride). Meanwhile, patient's vital signs were monitored by electrocardiography. After 800 ml of blood were transfused, the blood pressure increased to 110/70 mmHg. The blood tests (5 h after the first tests) showed that WBC was 7.93 × 10^9^/L, RBC 3.19 × 10^12^/L, HB 93 g/L, PLT 214 × 10^9^/L, and CRP 10.00 mg/L. On the 2nd day, besides the same volume of glucose and sodium chloride rehydration, antibiotics and analgesics, the patient was given infusion with 500 ml of multiple electrolytes rehydration (containing 2.63 g sodium chloride, 2.51 g sodium gluconate, 1.84 g sodium acetate, 0.185 g potassium chloride and 0.15 g magnesium chloride) and 100 ml of oral potassium chloride supplementation (containing 10 g potassium chloride). The 2nd day blood tests showed that WBC was 9.92 × 10^9^/L, RBC 3.43 × 10^12^/L, HB 106 g/L, PLT 192 × 10^9^/L, CRP 5.00 mg/L, K^+^ 3.90 mmol/L, Na^+^ 141 mmol/L, Cl^−^ 105 mmol/L. The 3rd day blood tests showed that WBC was 10.57 × 10^9^/L, RBC 3.22 × 10^12^/L, HB 102 g/L, PLT 212 × 10^9^/L, CRP 4.00 mg/L, K^+^ 4.70 mmol/L, Na^+^ 141 mmol/L, Cl^−^ 106 mmol/L. After about 2 weeks of treatment, the swelling of the patient's back obviously subsided and the ecchymosis area faded. Therefore, the treatment was effective and confirmed by CT examination on the 12th day after operation (Figure 3).

**Figure 4:**
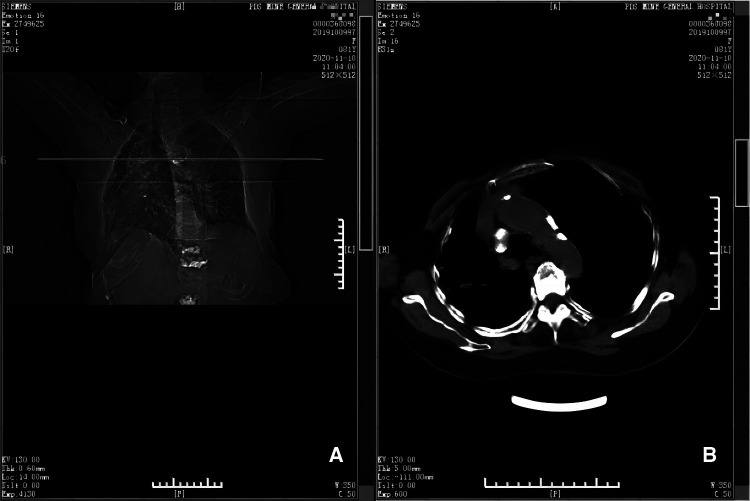
CT scan taken at 12th month postoperatively (panels **A** and **B**). Panel **A** depicts the position (horizontal line) of the CT scan shown in **B**, at the level of the 5th thoracic vertebra. Panel **B** shows there is no hematoma near the 5th thoracic vertebra and adjacent ribs.

**Figure 5:**
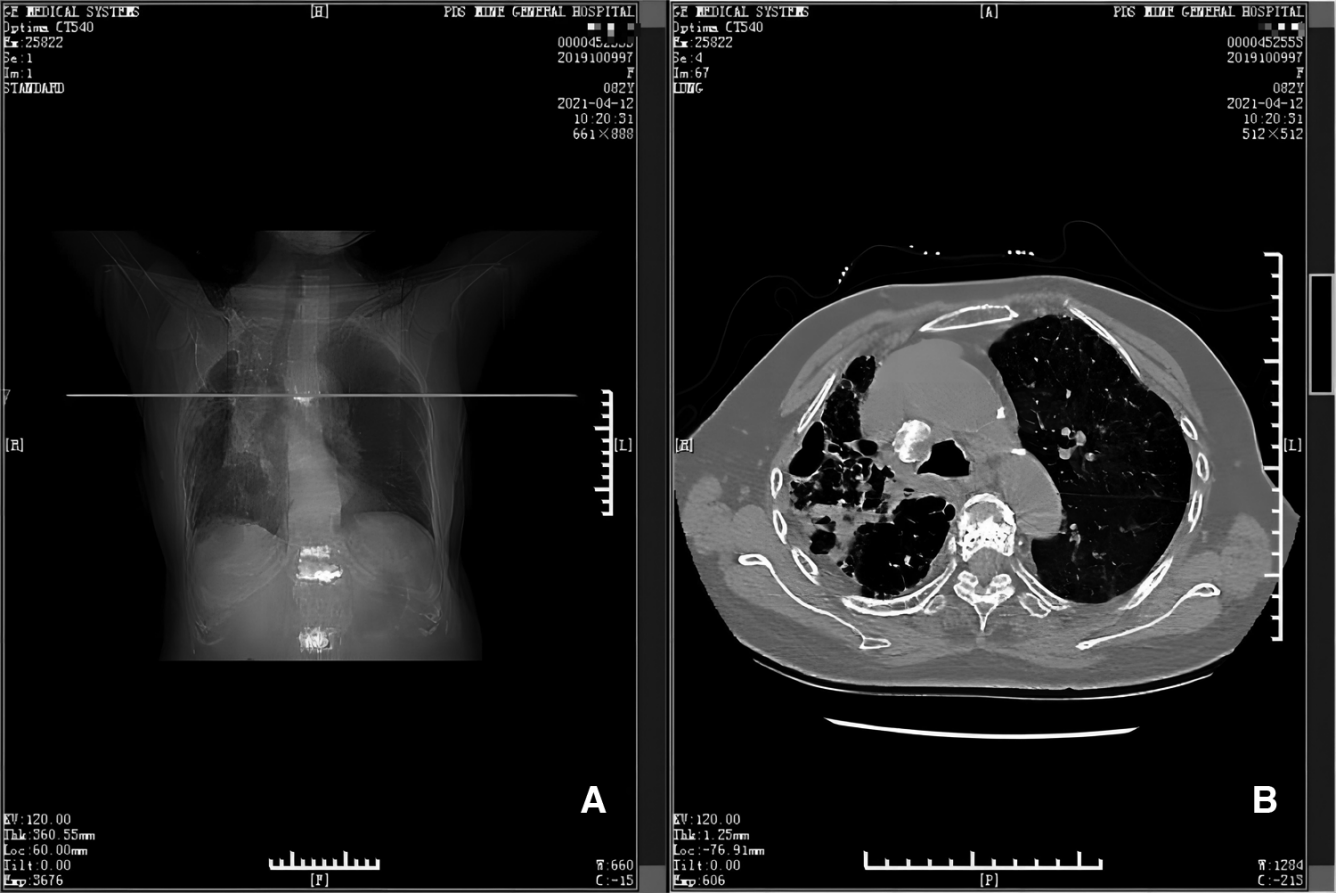
CT scan taken at 17th month postoperatively (panels **A** and **B**). Panel **A** depicts the position (horizontal line) of the CT scan shown in B, at the level of the 5th thoracic vertebra. Panel **B** shows there is no hematoma near the 5th thoracic vertebra and adjacent ribs.

An OVCF of the 3rd lumbar vertebra happened without visible reasons, on the 16th day after the 2nd surgery. PVP was performed again smoothly without any complications. Therefore, she was discharged on the next day. A follow-up of chest CT showed that there was no residual hematoma at 12th and 17th months postoperatively (Figures 4, 5). There was no recurrence during the follow-up.

## Discussion

Shock caused by massive hemorrhage after PVP is very rare. We found only two case reports by reviewing the literature of the past over 40 years, which detailed the treatment of vascular injury after PVP (22, 23). they both involved the lumbar spine, confirmed by angiography, and were treated by arterial embolization of uncontrolled vertebral artery bleeding. One case, an 84-year-old female, was re-hospitalized for pulsatile bleeding 10 days postoperatively following kyphoplasty of the 4th lumbar vertebra using a transpedicular approach (23). The other, a 73-year-old female, developed radiating pain and a tingling sensation in her left leg 2 h following PVP of the 2nd lumbar vertebra using an extrapedicular approach. Ultimately, the cause was attributed to bleeding, and 40 days postoperatively, 500 ml of liquified hematoma was aspirated from the retroperitoneum using ultrasound guidance (22).

In the present case, when formulating the surgical plan, it was considered that the patient had 4 OVCFs that needed surgical treatment. If all PVP were completed in one surgery, the operation time would become very long. This would greatly increase the risk of surgery, which was an adverse risk for patients. Therefore, we made a plan to complete all PVP in two times, which was approved by the patient and her family. First of all, the three OVCFs (T12/L1/L4) that were relatively easy to operate and had great influence on activities should be completed in the first operation. Then, the OVCF (T5) that was difficult to operate was completed in the second operation.

When shock occurred following PVP of the 5th thoracic vertebra, an emergency consultation was conducted. The chief physician participating in the consultation believed that there was a large amount of blood loss in the operation area, but the bleeding might be relatively limited. Therefore, a conservative treatment plan was adopted. Neither angiography nor vascular embolization was undertaken. She underwent rapid resuscitation with fluid and blood infusions, and an local ice bag was used to compress the hematoma. The local ice bag compressed the swollen part of back bleeding, aiming to enhance the effect of detumescence and hemostasis. The rationale for use of the local ice compression was to promote vasoconstriction, thereby reducing both bleeding and local swelling. Additionally, it could reduce the inflammatory reaction and provide an analgesic effect. Fortunately, the bleeding was successfully stopped, which avoided the need for vascular embolization. In addition, it was worth noting that, the effectiveness of local ice bag compression after surgery was usually limited to 48 to 72 h, and the compression range and duration of each ice bag could be appropriately adjusted according to the amount of blood exudation and the degree of swelling. Of course, local ice bag compression was only a part of the whole treatment process, and the treatment of shock cannot be separated from comprehensive treatment.

We analyzed the possible causes of massive hemorrhage after PVP in this case, and the explanations were as follows. First, although this was rarely encountered in the process of carefully guided needle insertion, needle puncture might cause potential vascular damage. The force applied during puncture might cause slight deviation of the path, or the patient's blood vessels might be in an abnormal position, which might lead to bleeding. Since angiography was not performed, the bleeding vessels were not clearly identified. Second, the patient's soft tissue around the 5th thoracic vertebra is relatively loose due to his thin body and old age. The retroperitoneal space could be greatly expanded, in which case it could hold a large amount of blood until the patient's vital signs, local pain or external evidence of bleeding were confirmed. Third, the patient had a slight hunchback and cannot lie flat for a long time, which limited the effectiveness of local ice bag compression. In addition, the effect of the ice bag on vasoconstriction might be weakened by the towel placed between the ice bag and the skin to reduce the freezing sensation.

When bleeding after PVP, we recommend the following measures that might be helpful. The vital signs and hematoma of the patient should be noticed in time to assess the severity of the bleeding. Infusion and blood transfusion should be carried out immediately to maintain effective blood pressure. Local ice bag compression should be carried out, which could play a certain role in hemostasis and detumescence. At the same time, maintaining communication with patients and their families was very important to prevent panic and obtain their cooperation. As shown in the previous case reports of lumbar hemorrhage, under the condition of continuous monitoring, if massive or continuous bleeding was diagnosed, emergency angiography should be performed and vascular embolization should be selected.

## Conclusion

PVP has been used by most doctors to treat OVCF because of its definite effect. Although the shock caused by massive hemorrhage after PVP of the 5th thoracic spine is very rare, it may endanger the patient's life and needs timely treatment.

## Data Availability

The original contributions presented in the study are included in the article, further inquiries can be directed to the corresponding author.
